# Innate-Like Control of Human iNKT Cell Autoreactivity via the Hypervariable CDR3β Loop

**DOI:** 10.1371/journal.pbio.1000402

**Published:** 2010-06-22

**Authors:** Gediminas Matulis, Joseph P. Sanderson, Nikolai M. Lissin, Maria B. Asparuhova, Gopal R. Bommineni, Daniel Schümperli, Richard R. Schmidt, Peter M. Villiger, Bent K. Jakobsen, Stephan D. Gadola

**Affiliations:** 1 Center for Experimental Rheumatology, University of Bern, Inselspital, Bern, Switzerland; 2 Division of Infection, Inflammation and Immunity, University of Southampton, School of Medicine, Sir Henry Wellcome and “Hope” Laboratories, United Kingdom; 3 Immunocore Ltd., Abingdon, United Kingdom; 4 Institute of Cell Biology, University of Bern, Bern, Switzerland; 5 Fachbereich Chemie, University of Konstanz, Konstanz, Germany; Whitehead Institute, United States of America

## Abstract

Invariant Natural Killer T cells (iNKT) are a versatile lymphocyte subset with important roles in both host defense and immunological tolerance. They express a highly conserved TCR which mediates recognition of the non-polymorphic, lipid-binding molecule CD1d. The structure of human iNKT TCRs is unique in that only one of the six complementarity determining region (CDR) loops, CDR3β, is hypervariable. The role of this loop for iNKT biology has been controversial, and it is unresolved whether it contributes to iNKT TCR:CD1d binding or antigen selectivity. On the one hand, the CDR3β loop is dispensable for iNKT TCR binding to CD1d molecules presenting the xenobiotic alpha-galactosylceramide ligand KRN7000, which elicits a strong functional response from mouse and human iNKT cells. However, a role for CDR3β in the recognition of CD1d molecules presenting less potent ligands, such as self-lipids, is suggested by the clonal distribution of iNKT autoreactivity. We demonstrate that the human iNKT repertoire comprises subsets of greatly differing TCR affinity to CD1d, and that these differences relate to their autoreactive functions. These functionally different iNKT subsets segregate in their ability to bind CD1d-tetramers loaded with the partial agonist α-linked glycolipid antigen OCH and structurally different endogenous β-glycosylceramides. Using surface plasmon resonance with recombinant iNKT TCRs and different ligand-CD1d complexes, we demonstrate that the CDR3β sequence strongly impacts on the iNKT TCR affinity to CD1d, independent of the loaded CD1d ligand. Collectively our data reveal a crucial role for CDR3β for the function of human iNKT cells by tuning the overall affinity of the iNKT TCR to CD1d. This mechanism is relatively independent of the bound CD1d ligand and thus forms the basis of an inherent, CDR3β dependent functional hierarchy of human iNKT cells.

## Abstract 2

T-cell receptor variability gives rise to a functional hierarchy of human invariant Natural Killer T-cells through a powerful effect on CD1d binding affinity, which is independent of CD1d ligands.

## Author Summary

Our immune system uses randomly modified T-cell receptors (TCRs) to adapt its discriminative capacity to rapidly changing pathogens. The T-cell receptor (TCR) has six flexible, variable peptide loops that make contact with antigens presented to them on the surface of other cells. Invariant Natural Killer T-cells (iNKT) are regulatory T-cells with a unique type of TCR (iNKT-TCR) that recognizes lipid antigens presented by specific MHC-like molecules known as CD1d. In human iNKT-TCRs, only one of the six loops, CDR3beta, is variable. By comparing how different human iNKT clones bind and react to different CD1d-lipid complexes we uncover the existence of a hierarchical order of the human iNKT cell repertoire in which strongly CD1d-binding clones are autoreactive while weak CD1d-binding clones are non-autoreactive. Direct measurements of iNKT-TCR binding to CD1d using surface plasmon resonance recapitulated this hierarchy at the protein level. The data show that variation in the CDR3beta loop conveys dramatic differences in human iNKT TCR affinity that are independent of the CD1d bound ligand. Thus the CDR3beta loop provides the structural basis for the functional hierarchy of the human iNKT repertoire. We postulate that during the life-course, CDR3beta-dependent asymmetrical activation of different human iNKT clones leads to a bias in the iNKT repertoire, and this could result in age-dependent defects of iNKT-mediated immune regulation in later life.

## Introduction

Invariant Natural Killer T (iNKT) cells are a conserved subset of highly potent and versatile T-cells which specifically recognize the non-polymorphic lipid-presenting molecule CD1d [UniprotKB P15813] [1]. iNKT cells co-express a unique T-Cell Receptor (iNKT TCR), which mediates recognition of CD1d, and the pan-NK receptor NKR-P1A (CD161). Human and mouse iNKT TCRs feature a homologous invariant TCRα chain, i.e. Vα24-Jα18 in humans and Vα14-Jα18 in mice. In addition, all human iNKT TCRs make use of a single TCR Vβ family, Vβ11, whereas mouse iNKT TCRs utilize several different TCR Vβ families.

The current paradox of iNKT biology lies in the fact that, despite their apparent innate-like simplicity, they can exert directly conflicting functions. On the one hand, several in vivo studies have demonstrated an essential role for iNKT cells in the induction and maintenance of immunological tolerance [2],[3]. Consistent with this, iNKT cells exert a protective role in animal models of spontaneous autoimmunity [4],[5], and numerical and functional defects of iNKT cells are observed in different human autoimmune diseases [6].

In contrast to these tolerogenic functions, iNKT cells can exert potent cytotoxic functions and contribute to host defense against tumors and various infectious pathogens [7],[8],[9]. Whether different subsets of iNKTs are involved in these opposed roles or whether individual iNKT clones fulfill both of these functions under different conditions is unknown. Several mechanisms underpin iNKT activation during host defense, such as TLR [10],[11],[12] and PPAR-γ activation [13], co-stimulatory molecule signaling [14], and inflammatory cytokines [15],[16]. However, it is unknown how iNKT cells are induced to mediate their tolerogenic functions under non-inflammatory conditions.

Some iNKT clones exhibit substantial activation in response to CD1d-expressing antigen-presenting cells in the absence of exogenous antigens. This autoreactive function is essential for both iNKT selection [17] and tolerogenic activity [18]. While iNKT TCR binding to CD1d is absolutely required [19], the mechanistic basis of iNKT cell autoreactivity is largely unresolved. In particular, the importance of specific CD1d-presented endogenous lipid antigens for the autoreactive interaction of the iNKT TCR with CD1d is contentious.

Studies in mice have suggested that the iNKT repertoire displays clonal heterogeneity with regard to recognition of weaker stimulatory lipid antigens, such as the α-galactosylceramide analogue OCH. These differences can be explained by the differential Vβ family usage in mouse iNKT TCRs [20],[21],[22]. However, human iNKT TCRs use a single Vβ family and so the short hypervariable complementarity determining region (CDR3β) loop in human iNKT TCRs is their only truly adaptive element. It is not known whether this is sufficient to endow the human iNKT TCR with meaningful ability to discriminate a diverse range of human CD1d-presented antigens.

Here we examined a large panel of human iNKT cell lines and clones for their binding to different CD1d-ligand tetramers and related this both to the affinity of their TCRs to different CD1d-ligand complexes and to their functional recognition of diverse antigens. The results presented here demonstrate that variations in the CDR3β loop have a profound, antigen-independent, impact on the iNKT TCR's affinity to CD1d and on iNKT cell autoreactive function.

## Results

### OCH-CD1d Tetramers Reveal Broad Heterogeneity of K7-CD1d Tetramer Positive Human iNKT Cells

Previous studies have shown that the CDR3β loop is dispensable for the ability of human iNKT cells to strongly react to the α-galactosylceramide antigen KRN7000 (K7), a xenobiotic glycolipid which can be presented to iNKT cells by CD1d. In fact, K7-CD1d tetramer staining does not allow discrimination of different human iNKT cell subsets by flow cytometry. We hypothesized that CD1d-tetramers loaded with weaker antigens might be better able to reveal the existence of CDR3β-dependent variation among human iNKT cells.

**Figure 1:**
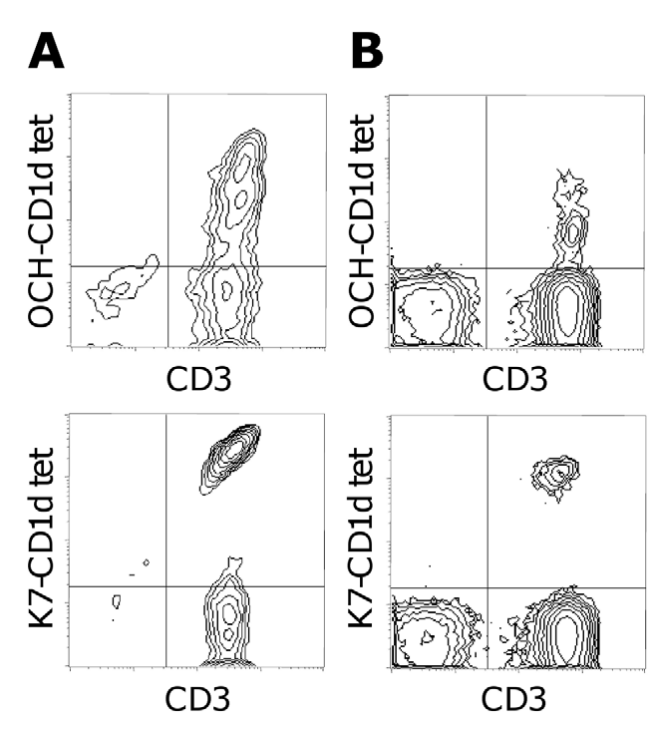
Distinct iNKT cell subpopulations revealed by OCH-CD1d tetramer staining. OCH- and K7-CD1d tetramer stainings of (A) a representative K7-stimulated human iNKT line after 14 d in vitro culture and (B) a healthy human volunteer's PBMC ex vivo are shown. While K7-CD1d tetramer staining identifies a single homogeneous population of iNKT cells (upper row), OCH-CD1d tetramer staining reveals the presence of different distinct iNKT populations within these samples (lower row).

Therefore, we first examined whether different human iNKT subsets could be segregated by their binding to CD1d tetramers that were loaded with the synthetic iNKT partial agonist antigen OCH. For this purpose, polyclonal iNKT lines, generated from healthy donors by in vitro stimulation with K7, were tested for their binding to both K7- and OCH-CD1d tetramers. In all of these lines, K7-CD1d tetramers stained a single, clearly distinct, homogeneous, and strongly fluorescent population of iNKT lymphocytes (Figure 1A). In contrast, staining of the same lines with OCH-CD1d tetramers revealed a considerable degree of variation in fluorescence, suggesting the presence of distinct iNKT subpopulations (Figure 1A). Importantly, similar qualitative differences between K7- and OCH-CD1d tetramer staining of iNKT cells could also be observed ex vivo (Figure 1B), indicating that these differences were not due to an artifact of previous in vitro stimulation with K7. In order to examine whether the broadly heterogeneous OCH-CD1d tetramer staining of human iNKT cells resulted from stable clonal variation or from transient changes in TCR expression levels, we generated a large panel of “K7/OCH-naïve” human iNKT cell clones and lines. For this purpose, Vα24+/Vβ11+ T cells were directly sorted ex vivo from healthy human donors and expanded using the non-specific T cell mitogen phytohaemagglutinin. Ninety-seven different human Vα24+/Vβ11+ T cell lines and 256 Vα24+/Vβ11+ T cell clones from 13 different healthy donors were established and analyzed by flow cytometry with K7- and OCH-CD1d tetramers.

**Figure 2:**
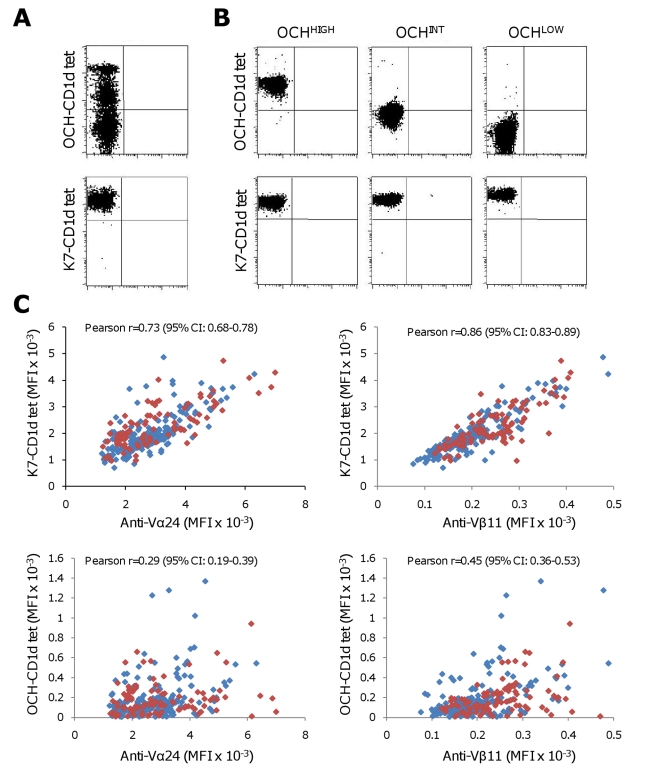
Clonal variation in OCH-CD1d tetramer binding by human iNKT cells is not related to TCR expression levels. Flow cytometric analysis of one representative CD4+ human Vα24+/Vβ11+ iNKT line (A) and three representative CD4+ human Vα24+/Vβ11+ iNKT clones from different donors (B) demonstrates clonal variation in binding to OCH-CD1d (upper row), but not K7-CD1d (lower row) tetramers. (C) K7- and OCH-CD1d tetramer staining in pure human iNKT lines (*n* = 68) and clones (*n* = 256) was related to expression levels of iNKT TCR Vα24 and Vβ11. The intensity (MFI) of K7- but not OCH-CD1d tetramer staining was strongly associated with Vα24 and Vβ11 expression, as determined by Pearson correlation analysis, but not with CD4+ (blue markers) or CD4−CD8− double negative (red markers) phenotype.

All Vα24+/Vβ11+ T-cell clones and lines showed bright, homogeneous staining with K7-tetramers (Figure 2), thereby confirming them as iNKT cells. Individual iNKT clones showed modest variation, up to 6-fold, in K7-CD1d tetramer mean fluorescence intensity (MFI). In contrast, multiple iNKT cell subpopulations with differing fluorescence intensities were revealed by OCH-CD1d tetramer staining in 31 of the 97 iNKT lines (Figure 2A), thereby mirroring the above described findings in K7 stimulated iNKT lines. As expected, all 256 iNKT clones stained homogeneously with OCH-CD1d tetramers. However, substantial differences, up to 200-fold, in OCH-CD1d tetramer MFI were observed between individual clones (Figure 2B). Based on the observed large differences in OCH-CD1d tetramer MFI, the 256 human iNKT clones were categorized as OCH^HIGH^ (MFI>300; *n* = 41), OCH^INT^ (MFI>50 and <300; *n* = 164), or OCH^LOW^ (MFI<50; *n* = 51).

Importantly, the differences in OCH-CD1d tetramer staining could not be explained by differences in either TCR or CD4 co-receptor expression. Whereas K7-CD1d tetramer binding significantly correlated with surface expression levels of the Vα24 and Vβ11 TCR chains, no such association was observed for OCH-CD1d tetramer staining (Figure 2C). Furthermore, CD4 co-receptor usage was not related to the intensity of the iNKT clones' OCH or K7-CD1d tetramer staining (unpublished results).

The results of these experiments revealed that the human iNKT repertoire is broadly heterogeneous with regard to the ability of individual clones to bind OCH-CD1d tetramers, independent of either CD4 co-receptor or TCR expression levels.

### Human OCH^HIGH^ and OCH^LOW^ iNKT Cells Exhibit Differential Binding to CD1d Molecules Presenting β-Glycosylceramide

**Figure 3:**
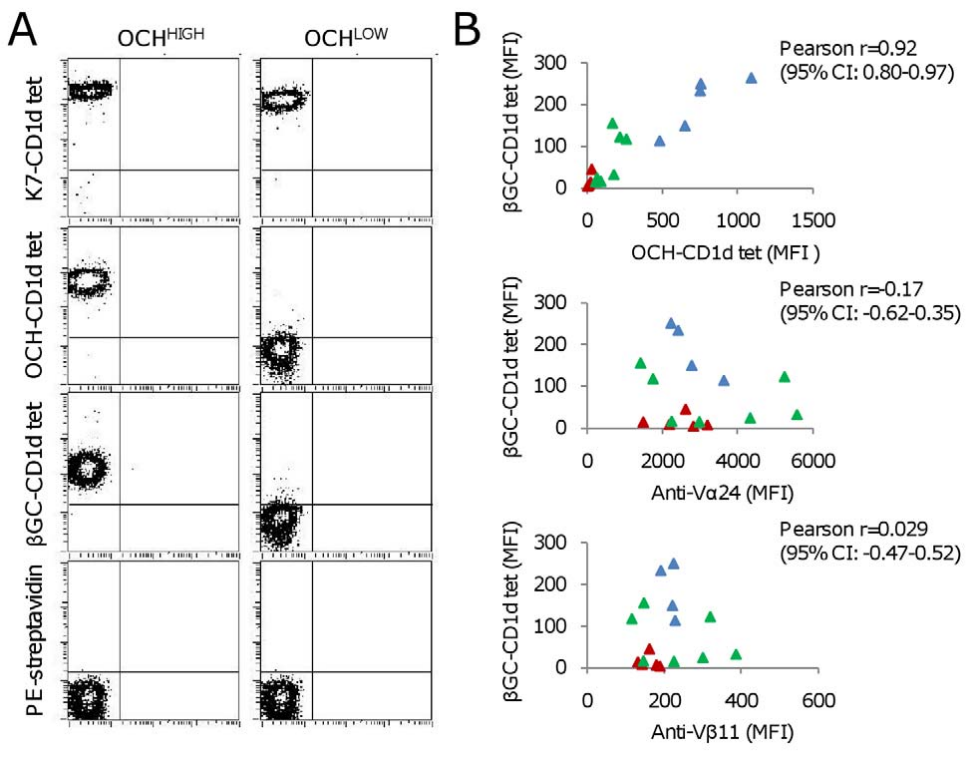
Differential binding of OCH^HIGH^ and OCH^LOW^ iNKT cells to βGC-CD1d tetramers. Ex vivo sorted human Vα24+/Vβ11+ iNKT clones were stained with different, α- or β-glycosylceramide loaded CD1d-tetramers. (A) A representative pair of CD4+ OCH^HIGH^ and OCH^LOW^ iNKT clones from one donor is shown. βGC-CD1d tetramers only stained OCH^HIGH^ but not OCH^LOW^ iNKT clones above background (as determined by PE-streptavidin binding). TCR Vα24 and Vβ11 surface expression levels for the two clones shown were very similar (for PE-conjugated anti-Vα24, MFI 2673 (OCH^HIGH^) and 2710 (OCH^LOW^); for FITC-conjugated anti-Vβ11, MFI 106 (OCH^HIGH^) and 97 (OCH^LOW^)). (B) βGC-CD1d tetramer staining intensity (MFI) of a panel of OCH-LOW (red markers), OCH-INT (green markers), and OCH-HIGH (blue markers) iNKT clones showed almost linear correlation with OCH-CD1d tetramer staining, but no correlation with either Vα24 or Vβ11 surface expression.

The above results indicated that clonally distributed qualitative differences in iNKT TCRs were responsible for the considerable variation in OCH-CD1d tetramer binding. However, differences in iNKT TCR mediated recognition of an unnatural compound like OCH would be physiologically irrelevant if they simply reflected random differences in OCH-specific antigen selectivity. To explore this possibility, 18 iNKT clones of broadly varying OCH-CD1d MFI were tested for their ability to bind CD1d tetramers loaded with the common mammalian glycolipid β-glycosylceramide (βGC). These 18 iNKT clones displayed significant variation, up to 50-fold, in βGC-CD1d tetramer staining (Figure 3A). Importantly, a strong association was evident between OCH-CD1d tetramer staining and βGC-CD1d tetramer staining, while no correlation was seen between βGC-CD1d tetramer staining and Vα24 TCR chain surface expression (Figure 3B). These results demonstrated that the observed broad variation in OCH-CD1d tetramer binding between individual human iNKT clones was not simply due to their antigen selectivity but was a reflection of a general variability in human iNKT TCR binding to CD1d loaded with weak antigenic lipids. Furthermore, they indicated that OCH-CD1d tetramer binding can act as a surrogate marker for human iNKT cell recognition of endogenous CD1d antigens.

### The Hypervariable CDR3β Loop Has a Strong Effect on the Affinity of Human iNKT TCRs to CD1d Presenting Either α- or β-Anomeric Glycolipids

**Figure 4:**
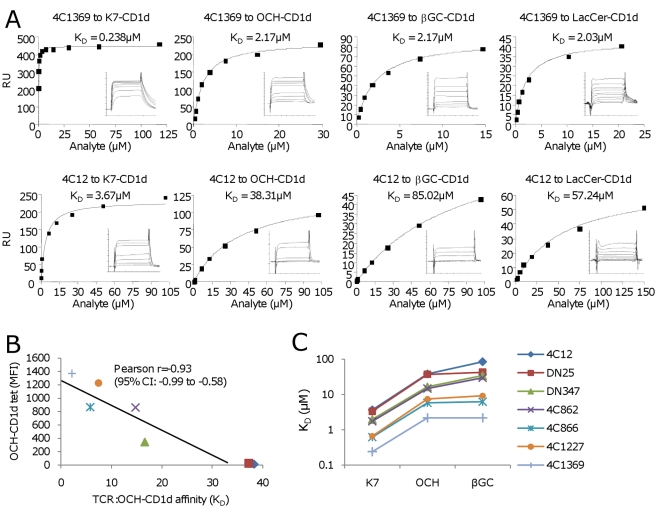
The CDR3β loop strongly impacts on human iNKT TCR affinity to CD1d, independent of the CD1d-bound ligand. (A) Binding of two recombinant human iNKT TCRs, one OCH^HIGH^ (4C1369) and one OCH^LOW^ (4C12), to K7-, OCH-, βGC-, and LacCer-CD1d at equilibrium is shown (see also panel C and Table 2). (B) The affinity of the seven recombinant iNKT TCRs to OCH-CD1d, as determined by SPR, was linearly related to the staining intensity (MFI) of the original iNKT clone with OCH-CD1d tetramers. (C) The seven recombinant human iNKT TCRs followed a strict hierarchy of binding to ligand-CD1d complex, which was not affected by the specific CD1d-bound ligand. These iNKT TCRs differed only with regard to their CDR3beta sequence (Table 1).

Based on the above results we hypothesized that the observed substantial differences in tetramer staining between OCH^HIGH^ and OCH^LOW^ iNKT clones resulted from significant variations in TCR:CD1d binding affinity. As expected, sequencing of the TCR Vα24 and Vβ11 chains demonstrated the usage of the known invariant Vα24-Jα18 rearrangement in all clones, while Vβ11 in these clones was rearranged with several different Jβ families, resulting in highly variable CDR3β sequences. This indicated that, in human iNKT TCRs, structural differences of the CDR3β loop have a substantial impact on iNKT TCR binding to CD1d. To test this in a cell-free system we cloned the extracellular domains of the TCR Vβ11 chains from a panel of seven OCH^HIGH^ and OCH^LOW^ iNKT cell clones (Table 1), as well as the invariant TCR Vα24 chain from one iNKT clone, and used them to generate soluble Vα24/Vβ11 iNKT TCRs. Binding of these recombinant iNKT TCRs to K7-, OCH-, as well as βGC- and lactosylceramide (LacCer-) loaded recombinant human CD1d complexes was measured using surface plasmon resonance (Figure 4A; Table 2).

**Table 1:** Characteristics of 7 different human iNKT TCRs.

iNKT	OCH-tet (MFI)	CD4/DN	Vα	Jα	Vβ	Jβ	Vβ seq.	N-(Dβ)-N	Jβ Sequence
4C12	12	CD4	24	18	11	1–5	CASS	GDRRQGAH	QPQHFGDGTRLSIL
DN25	25	DN	24	18	11	2–7	CAS	ARGVN	EQYFGPGTRLTVT
DN347	347	DN	24	18	11	1–1	CASS	AMD	TEAFFGQGTRLTVV
4C862	862	CD4	24	18	11	1–1	CASS	DQN	TEAFFGQGTRLTVV
4C866	866	CD4	24	18	11	2–7	CAS	TGASGT	YEQYFGPGTRLTVT
4C1227	1227	CD4	24	18	11	1–3	CASSE	PS	SGNTIYFGEGSWLTVV
4C1369	1369	CD4	24	18	11	2–5	CASSE	FGGTERT	QETQYFGPGTRLLVL

DN, double negative (CD4-CD8αβ-); Vα, Vβ, Variable α and β family; Jα, Jβ, Junctional α and β regions; N, N-region; Dβ, diversity region.

**Table 2:** Binding of 7 human iNKT TCRs to different CD1d/ligand complexes.

iNKT	K7-CD1d		OCH-CD1d		βGC-CD1d	
	K_D_ (µM)	T½ (sec)	K_D_ (µM)	T½ (sec)	K_D_ (µM)	T½ (sec)
4C12	3.67±0.85	0.99±0.03	38.31±1.50	0.71±0.03	85.01±5.96	ND
DN25	3.34±0.17	0.92±0.03	37.27±1.62	ND	42.43±1.88	ND
DN347	1.99±0.17	1.01±0.06	16.64±0.65	0.59±0.01	34.50±2.74	ND
4C862	1.75±0.13	1.02±0.04	14.80±0.59	0.63±0.05	29.65±3.69	ND
4C866	0.62±0.03	2.24±0.05	5.82±0.37	1.13±0.13	6.26±0.91	1.42±0.16
4C1227	0.66±0.06	2.55±0.08	7.45±0.59	1.10±0.07	9.19±1.61	ND
4C1369	0.24±0.01	12.38±0.36	2.17±0.16	4.78±0.55	2.17±0.13	4.36±0.73

K_D_, dissociation constant; T**½**, dissociation half-time; ND, not determined. All values given ± standard deviation.

The results of these experiments showed a striking variation, up to 40-fold, between the different iNKT TCRs in their binding affinity (K_D_) to a given ligand-CD1d complex (for K7-CD1d, K_D_: 0.24–3.67 µM; for OCH-CD1d, K_D_: 2.17–38.3 µM; for βGC-CD1d, K_D_: 2.17–85 µM; for LacCer-CD1d, K_D_: 2.1–54 µM; see Table 2). These findings clearly showed that the CDR3β loop of human iNKT TCRs can strongly impact on their binding to ligand-CD1d complexes.

Importantly, the binding affinities of all seven recombinant iNKT TCRs to OCH-CD1d strongly correlated with the OCH-CD1d tetramer staining (MFI) of their corresponding original iNKT clones (Figure 4B). Moreover, the binding affinity of a given iNKT TCR to OCH-CD1d also correlated closely with its affinity to either βGC- or K7-CD1d (Figure 4C). Therefore, the wide variation in affinity between our seven human iNKT TCRs contrasted to the lack of variation in antigen selectivity. In other words, the CDR3β loop of human iNKT TCRs modulated the overall binding affinity to different human ligand-CD1d complexes irrespective of the bound ligand.

Based on these findings we hypothesized that the TCRs of OCH^HIGH^ iNKT clones could also mediate enhanced functional recognition of endogenous ligand-CD1d complexes. We tested this hypothesis by comparing autoreactive responses of OCH^HIGH^ and OCH^LOW^ iNKT clones to CD1d-expressing antigen-presenting cells.

### Autoreactive Functions of Human iNKT Cells Correlate with Their OCH-CD1d Binding

**Figure 5:**
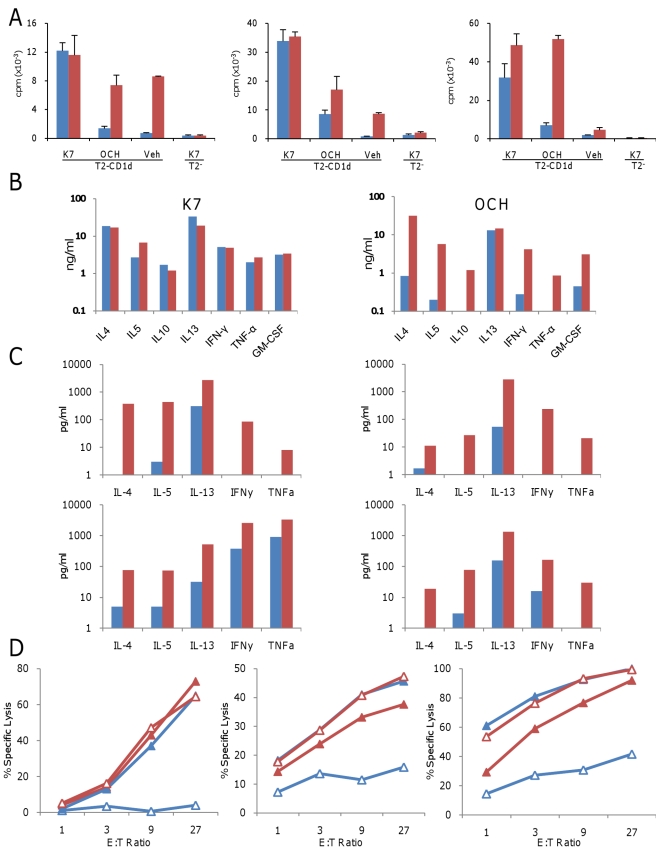
Differential autoreactive functional responses by human OCH^HIGH^ and OCH^LOW^ iNKT clones. Matched pairs of human OCH^HIGH^ (red columns and markers) and OCH^LOW^ (blue columns and markers) iNKT clones were compared for their ability to proliferate, secrete cytokines, and exhibit cytotoxicity in response to lipid-pulsed or endogenous lipid presenting CD1d-positive antigen presenting cells. (A) Proliferation of three representative pairs of OCH^HIGH^ and OCH^LOW^ iNKT clones from different healthy donors in response to K7-, OCH-, or vehicle-pulsed human CD1d-expressing T2 cells (T2-CD1d) or to K7-pulsed CD1d negative T2 cells (T2-) is shown. OCH^HIGH^ clones consistently displayed greater proliferation than OCH^LOW^ clones in response to OCH or vehicle pulsed T2-CD1d. cpm, counts per minute. Mean values ± s.e.m. are shown. (B) Cytokine secretion profiles of a representative pair of matched OCH^HIGH^ and OCH^LOW^ iNKT clones in response to the strong agonist ligand K7 and the partial agonist ligand OCH, presented by T2-CD1d, are shown. OCH^HIGH^ iNKT clones exhibited much stronger cytokine secretion than OCH^LOW^ iNKT cells in response to OCH-pulsed T2-CD1d, while cytokine secretion was similar for both in response to K7-pulsed T2-CD1d. (C) Autoreactive cytokine release in response to T2-CD1d in the absence of added exogenous ligands is shown for four matched pairs of OCH^HIGH^ and OCH^LOW^ iNKT clones. OCH^HIGH^ but not OCH^LOW^ iNKT clones consistently exhibited substantial autoreactive cytokine secretion. (D) Specific lysis of K7- (filled markers) and OCH- (unfilled markers) pulsed T2-CD1d targets is shown for three matched pairs of OCH^HIGH^ and OCH^LOW^ iNKT clones from different donors.

We directly compared the extent of proliferation, cytokine secretion, and cytotoxicity of human OCH^HIGH^ and OCH^LOW^ iNKT cells in response to CD1d expressing human cell lines presenting either endogenous or specific exogenous (“pulsed”) glycolipids. Because functional responses of iNKT cells might change during long term in vitro culture, we compared different donor-matched pairs of OCH^HIGH^ and OCH^LOW^ iNKT cell clones with identical in vitro history, i.e. each pair was sorted from a given donor 3 wk prior to the experiment and kept under identical cell culture conditions until the day of the experiment. The selected clones were all CD4+ and were additionally matched for TCR expression levels. For all pairs, OCH^HIGH^ iNKT clones exhibited significantly greater proliferation than OCH^LOW^ iNKT clones in response to either unpulsed or OCH-pulsed T2-CD1d lymphoblasts. In contrast, when T2-CD1d were pulsed with the strong agonist ligand K7, both OCH^HIGH^ and OCH^LOW^ iNKT clones proliferated vigorously, and to similar extent (Figure 5A).

Next, we measured CD1d-dependent secretion of a panel of cytokines by OCH^HIGH^ and OCH^LOW^ iNKT clones. The OCH^HIGH^ iNKT clones secreted considerably greater quantities of cytokines than their OCH^LOW^ counterparts in response to either unpulsed or OCH-pulsed T2-CD1d cells (Figure 5B, C), while no significant differences in cytokine secretion were observed between OCH^HIGH^ and OCH^LOW^ iNKT clones upon stimulation with K7-pulsed T2-CD1d cells. A general Th0-type cytokine secretion pattern was observed in response to stimulation with either K7 or OCH, while a Th1 pattern was often produced by autoreactive stimulation of OCH^HIGH^ iNKT (Figure 5C). Although most OCH^LOW^ iNKT clones did not exhibit autoreactive cytokine release, two OCH^LOW^ iNKT clones reproducibly secreted significant amounts of IL-13 and either IL-4 or IL-5, but no IFNγ or TNF-α, while one OCH^LOW^ iNKT clone secreted measurable amounts of IFNγ and TNF-α, but no Th2 cytokines.

None of the tested iNKT clones secreted detectable amounts of cytokines in response to CD1d-deficient T2-lymphoblasts, and blocking of surface CD1d molecules on T2-CD1d by the monoclonal antibody CD1d42 effectively prevented autoreactive secretion of cytokines by OCH^HIGH^ or OCH^LOW^ iNKT cells (unpublished data). Therefore, autoreactive cytokine secretion by these iNKT clones was wholly dependent on their recognition of surface CD1d.

**Figure 6:**
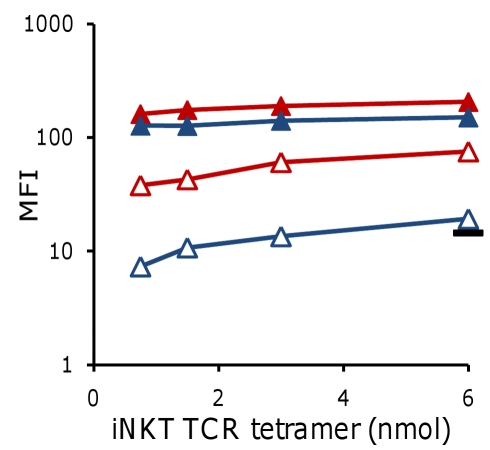
Differential binding of OCH^HIGH^ and OCH^LOW^ iNKT clone derived TCR tetramers to endogenous lipid presenting CD1d molecules. PE-conjugated recombinant iNKT TCR tetramers derived from OCH^HIGH^ (4C1369; red lines) and OCH^LOW^ (4C12; blue lines) iNKT clones, at increasing concentrations, were used to stain T2-CD1d lymphoblasts. Clear staining of vehicle-pulsed T2-CD1d (unfilled markers) was only seen with the OCH^HIGH^ TCR tetramer, whereas both iNKT TCR tetramers strongly bound to K7-pulsed T2-CD1d (filled markers). The black bar shows background staining of T2- cells with iNKT TCR tetramers.

Finally, in Cr^51^ release assays, OCH-pulsed T2-CD1d were much more efficiently killed by OCH^HIGH^ iNKT clones than their corresponding OCH^LOW^ iNKT clones (Figure 6D). In contrast, K7-pulsed T2-CD1d were efficiently lysed by both OCH^HIGH^ and OCH^LOW^ iNKT clones, whereas neither OCH^HIGH^ nor OCH^LOW^ iNKT clones showed relevant cytotoxicity towards unpulsed T2-CD1d lymphoblasts.

Together, these results demonstrated that OCH-CD1d tetramer staining allows for identification of distinct human OCH^HIGH^ and OCH^LOW^ iNKT clones, which exhibit differential functional ability to respond to endogenous ligand-CD1d complexes. The above results indicated that the autoreactive potential of human iNKT clones is governed by the affinity of their iNKT TCR to CD1d, and therefore the structure of their CDR3β loop.

### TCRs from OCH^HIGH^ but not OCH^LOW^ Human iNKT Subsets Bind to Endogenous CD1d-Ligand Complexes

In order to test our hypothesis that OCH^HIGH^ and OCH^LOW^ iNKT TCRs differed in their binding to endogenous ligand-CD1d complexes, we generated soluble fluorescent iNKT TCR-tetramers derived from an autoreactive OCH^HIGH^ iNKT clone and a non-autoreactive OCH^LOW^ iNKT clone. As shown in Figure 6, both iNKT TCR tetramers bound well to K7-pulsed T2-CD1d. In contrast, only the OCH^HIGH^-derived iNKT TCR tetramer was able to effectively stain unpulsed T2-CD1d. These results further substantiated our hypothesis that autoreactive recognition of CD1d by human iNKT cells is primarily determined by the structure of their iNKT TCRs' CDR3β loop.

All together, these studies demonstrated that the human iNKT cell repertoire exhibits considerable clonally distributed CDR3β-dependent differences in overall TCR affinity to CD1d, irrespective of the bound ligand, and that these inherent structural differences control iNKT autoreactive activation.

## Discussion

iNKT cells are a conserved subset of highly potent regulatory T cells at the innate-adaptive interface. The hallmark of human iNKT cells is their unique TCR, which is composed of an invariant TCR Vα24-Jα18 alpha chain and a semi-invariant TCR Vβ11 chain. The only variable, and therefore potentially adaptive, element in human iNKT TCRs is their hypervariable CDR3β loop. The results of the present study demonstrate for the first time, to our knowledge, that the structure of the hypervariable CDR3β loop in human iNKT TCRs exerts a strong impact on CD1d binding and is a key determinant of iNKT cell autoreactivity. The magnitude of the effect of CDR3β variations on human iNKT TCR:CD1d binding observed here was unexpected as previous studies with mouse iNKT TCRs have reported only minor effects of CDR3β mutations on CD1d binding. Furthermore, they strongly suggest that CDR3β loops in autoreactive iNKT TCRs make functionally important direct protein-protein contacts with human CD1d, rather than contacts with CD1d-bound ligands, thereby affecting overall affinity rather than antigen specificity.

The role of the hypervariable CDR3β loop in human iNKT TCRs is currently unresolved. It is dispensable for binding to CD1d molecules that are loaded with the strong agonist ligand K7, and hence K7-CD1d tetramers do not support subset differentiation of human iNKT cells. Consistent with this, the recently solved structures of one human and two mouse iNKT TCR:K7-CD1d co-crystals have found no relevant contacts between CDR3β and the K7-CD1d complex [20],[23]. In contrast, recent mutagenesis studies have indicated that the CDR3β loop of mouse iNKT TCRs may exert some impact on the affinity to CD1d, particularly when CD1d was loaded with weaker antigens [24],[25],[26].

We found that human iNKT cells were surprisingly heterogeneous in their binding to CD1d tetramers loaded with the partial agonist ligand OCH, which is a synthetic analogue of K7. Up to 200-fold differences in OCH-CD1d tetramer staining were observed between individual iNKT clones, independent of variations in TCR expression. The same clones exhibited only modest differences in K7-CD1d tetramer staining, which could largely be explained simply by variations in TCR expression. Importantly, we found that the clonal variation in OCH-CD1d tetramer binding was directly related to OCH-CD1d dependent functional responses, while no such linkage was observed between K7-CD1d tetramer staining and K7-dependent functional iNKT activation. These data underpinned the notion that the five germline encoded CDR loops in human iNKT TCRs, i.e. CDR1α-3α and CDR1β-2β, are sufficient for effective iNKT cell interaction with K7-CD1d [26]. Importantly, they strongly indicated that productive iNKT TCR interactions with OCH-CD1d require additional binding energy provided by certain CDR3β loop structures. We tested this hypothesis by directly measuring the binding of K7- and OCH-CD1d complexes to a panel of seven recombinant human iNKT TCRs, which were derived from selected OCH^HIGH^ and OCH^LOW^ iNKT clones. These recombinant iNKT TCRs differed only in their CDR3β structure. The results of these experiments demonstrated that the broad clonal heterogeneity in OCH-CD1d tetramer staining is indeed directly determined by the iNKT clones' TCRs binding affinities to OCH-CD1d, and hence the structure of the CDR3β loop. Conversely, while all tested recombinant iNKT TCRs bound approximately 10-fold better to K7-CD1d than to OCH-CD1d, the fold-differences in affinity between the strongest and the weakest binding iNKT TCRs were similar for binding to either OCH- or K7-CD1d. Together with the above discussed tetramer-based and functional studies, this indicates that the synthetic CD1d ligand K7 pushes the interaction between human CD1d and iNKT TCRs beyond a physiological range. This is consistent with numerous in vivo and in vitro studies which showed that K7 induces concurrent massive iNKT cell secretion of TH1-, TH2-, and TH17-type cytokines, whereas OCH causes a clearly TH2-biased cytokine secretion pattern [27]. Also, addition of K7 to mouse fetal thymic organ cultures leads to effective deletion of iNKT cells [28], and K7 stimulation induces a prolonged anergy in iNKT cells [29], which supports the view that K7 is not a physiological ligand for iNKT cells. Hence, a full understanding of the biological role of CDR3β loop polymorphism will require more studies with weaker agonistic antigens, and the results of this study suggest that OCH is a good surrogate for endogenous weak agonist antigens.

There are two competing models to explain how differences in CDR3β loop structure could translate into variations of weak antigen recognition. In an “antigen-dependent” or “adaptive” model, the CDR3β loop bestows upon iNKT cells a degree of lipid selectivity by controlling iNKT TCR affinity to CD1d in a lipid antigen-specific manner. Alternatively, in an “antigen-independent” or “innate-like” model, the CDR3β loop structure modulates iNKT TCR binding affinity to CD1d via protein-protein interactions. This model would allow higher, but not lower, affinity TCR structures to recognize CD1d molecules presenting weaker lipid antigens but, crucially, without differential patterns of lipid antigen selectivity between iNKT TCRs of similar CD1d affinity. In other words, this model predicts that the inherent CDR3β sequence in a given human iNKT clone would determine its iNKT TCR's general ability to bind to diverse ligand-CD1d complexes. An important corollary of this would be a fixed hierarchy of high and low affinity iNKT clones. A prediction arising from this model would be that iNKT cells lack the ability to develop immunological memory to specific pathogens, which is a hallmark of adaptive immunity. Although iNKT TCRs clearly belong to the broader family of rearranged, and therefore “adaptive,” TCRs and BCRs, their limited structural diversity and lack of antigen-selectivity, as proposed by this model, are strongly reminiscent of innate immune receptors.

In order to test which of the two above models best explains the observed CDR3β-dependent variation in iNKT TCR binding to OCH-CD1d, we examined recognition of two β-linked glucosylceramides, βGC and LacCer, by a panel of iNKT TCRs. K7 and OCH are α-linked monosaccharide glycosylceramides and are not expressed in mammals, whereas βGC and LacCer are natural β-linked glycosylceramides of mammalian cell membranes. The different configurations of α- and β-anomeric glycolipids enforce substantial differences in the orientation of their glycosyl headgroups when presented by CD1d [30],[31]. Therefore, if the substantial variation in iNKT TCR affinity to OCH-CD1d observed in our study was mainly a function of clonal variation in lipid antigen specificity, as predicted by the “adaptive” model, there should be no association between an individual iNKT TCR's affinity to OCH-CD1d and its affinity to either βGC-CD1d or LacCer-CD1d. However, the results of the present study strongly support the “innate” model: βGC-CD1d tetramer binding to human iNKT clones correlated in a linear fashion with OCH-CD1d tetramer binding, and our binding studies with several different soluble iNKT TCRs demonstrated that the CDR3β loop of human iNKT TCRs strongly modulated the overall binding affinity to different human ligand-CD1d complexes, independent of the bound ligand.

CDR3β loop hypervariability of human iNKT TCRs therefore strongly impacts on overall affinity to CD1d but does not exert a relevant effect on antigen selectivity. The powerful effect of natural CDR3β variations on human iNKT TCR:CD1d affinity observed in our study was unexpected as previous iNKT TCR mutagenesis studies in mice have suggested only a weak impact of CDR3β structure on iNKT TCR binding affinity [24],[25],[26]. Indeed, hybridomata expressing mouse iNKT TCRs with randomized CDR3β regions only displayed moderate variability in binding to K7-CD1d tetramers, and only very few TCRs were capable of interacting with CD1d presenting endogenous lipids [25].Furthermore, previously published iNKT TCR:CD1d co-crystal structures showed a negligible contribution of the CDR3β to the interaction [20],[23]. The apparent discrepancies between these studies and the current findings could indicate relevant species differences, as the mutagenesis studies have concentrated on mouse iNKT binding or else might reflect differences in study design: the only crystal structure study of human iNKT TCR:CD1d binding was limited to a single iNKT TCR of unknown weak antigen-CD1d affinity while the current study systematically screened a large panel of naturally occurring human iNKT clones. Interestingly, while the iNKT TCR used for the human co-crystal structure study displayed very limited contacts between its CDR3β loop and CD1d, a modeling exercise of TCR Vβ11 docking onto CD1d in the same study [23] pointed to a significant degree of plasticity of the CDR3β conformation. In particular, the CDR3β loop of one of our previously published CD1d-restricted Vα24− Vβ11+ TCRs, TCR 5E [32], could make significant contacts with the alpha-2 helix of human CD1d [23]. Consistent with this, a refolded hybrid TCR of the 5E Vβ11 chain and the invariant Vα24-Jα18 chain binds with high affinity to both CD1d/OCH and CD1d/βGC (unpublished data). Therefore, certain CDR3β loop structures can potentially facilitate the recognition of human CD1d loaded with weak ligands by providing additional binding energy to the TCR-CD1d interaction.

Sequence analysis of the CDR3β loops studied did not reveal any obvious correlations between CD1d binding affinity and either physicochemical properties of the loop as a whole or the position of specific residues within the sequence. This is not surprising, given the high degree of conformational flexibility of CDR loops.

The above described considerable binding affinities of some human iNKT TCRs to naturally occurring beta-anomeric glycolipids, i.e. βGC and LacCer, have important implications for the clonal distribution of iNKT autoreactivity. CD1d-dependent autoreactivity of iNKT cells, i.e. their CD1d-mediated activation in the absence of exogenous antigens, is likely to play important biological roles, but the molecular mechanisms determining iNKT autoreactivity have been unresolved. CD1d-dependent autoreactivity is observed in approximately 30% of mouse iNKT hybridomas[19], and studies in iNKT deficient and autoimmune prone mice have shown that autoreactive CD1d-recognition is required for iNKT selection and also iNKT-mediated immunological tolerance [15],[18],[33],[34]. However, much less is known about the role of CD1d-dependent iNKT autoreactivity in humans. Neonatal human iNKT cells exhibit an activated memory phenotype, indicating their in vivo recognition of CD1d molecules in the absence of exogenous ligands [35].

An “adaptive” model has been proposed to explain autoreactive activation of iNKT cells in mouse models of bacterial infection, and it was postulated that autoreactive murine iNKT cells specifically recognize *de novo* synthesized antigens, such as isogloboside 3 [36]. Consistent with this model, mouse CD1d requires endosomal trafficking to elicit autoreactive activation of murine iNKT cells, which suggests that processing of the ligand-CD1d complex is essential [37]. However, in contrast to mouse iNKT cells, human iNKT cell autoreactivity is not dependent on CD1d trafficking or endosomal acidification [38], again suggesting important species differences between mouse and human iNKT cell activation.

The antigen-independent “innate-like” model discussed above offers a simpler explanation for the clonally distributed iNKT autoreactivity. iNKT clones with higher overall iNKT TCR:CD1d affinity would have an intrinsically greater autoreactive potential than low affinity clones, and these differences in autoreactive potential would be independent of *de novo* synthesized CD1d-bound ligands. Autoreactive activation of iNKT clones in this model would still be controlled by local conditions, such as TLR signaling [12], CD1d expression [16], or cytokine expression [39]. High affinity iNKT clones would be capable of exerting autoreactive functions under physiological conditions, while low affinity iNKT clones would only be recruited under more pro-inflammatory conditions, e.g. during bacterial infections.

Our functional analyses of autoreactive activation of OCH^HIGH^ and OCH^LOW^ iNKT clones support the “innate-like” model. Firstly, autoreactive activation of several matched pairs of human iNKT clones was closely associated with their OCH-CD1d tetramer binding characteristics. Secondly, only iNKT TCR-tetramers generated from OCH^HIGH^ iNKT clones were able to bind to CD1d-expressing antigen-presenting cells in the absence of exogenous lipid. The above data therefore underpin the “innate-like” model, whereby the hypervariable CDR3β loop balances TCR binding affinity to CD1d protein, and hence the autoreactive potential of an iNKT clone, independent of the bound ligand.

The different activation thresholds of ex vivo sorted human OCH^HIGH^ and OCH^LOW^ iNKT clones shown herein suggest different in vivo functions of these subsets. For example, OCH^HIGH^ and OCH^LOW^ iNKT cells might differ in their ability to drive the formation of immature DCs and consequently in their capability to constitutively promote peripheral tolerance. Finally, it is intriguing to speculate that CDR3β-dependent asymmetrical activation of the human iNKT repertoire could, over time, skew the balance between OCH^HIGH^ and OCH^LOW^ iNKT clones, with ensuing consequences for iNKT-dependent functions in both host defense and immunological tolerance.

## Methods and Materials

### Generation of Human iNKT Cell Clones and Lines

Peripheral blood mononuclear cells (PBMC) were isolated from human peripheral venous blood by density gradient centrifugation (Ficoll-Hypaque; Amersham Pharmacia and Upjohn). The study was approved by the local ethics committee (KEK, Bern, Switzerland). All donors gave informed consent. Human iNKT clones and lines were generated by FACSVantage sorting of Vα24+/Vβ11+ T cells into round-bottomed 96-well plates. Sorted cells were stimulated with 1 µg/ml phytohaemagglutinin (Remel, USA) in the presence of autologous γ-irradiated (35Gy) PBMCs. Cells were grown in T cell growth medium (RPMI 1640, 2% human AB serum (SRK, CH), 10% fetal bovine serum (FBS), 0.1 mg/ml kanamycin, 1 mM sodium pyruvate, 1% non-essential amino acids, 1% L-glutamax, and 50 µM 2-mercaptoethanol (all from Gibco Invitrogen) and IL-2 (Proleukin, Chiron) 200 IU/ml). IL-2 concentration in the medium was gradually reduced to 20 IU/ml 3 wk after sorting.

### Flow Cytometry

The following fluorescent reagents were used to analyze human iNKT cells: PE-conjugated human CD1d tetramers loaded with either K7, OCH, βGC [40]; FITC-conjugated anti-human TCR Vβ11, PE-anti-human TCR Vα24, (Serotec, UK); PerCP-anti-CD3, FITC-anti-CD3, APC-anti-CD4, APC-anti-CD8, (BD Pharmingen). After addition of staining reagents, cells were incubated at 4°C for 45 min, washed twice in ice-cold PBS/1% FBS, and acquired on a four-color FACSCalibur flow cytometer (Becton Dickinson). Propidium iodide was used to exclude dead cells. Data were processed using CellQuest Pro software (BD Biosciences, USA). Staining with PE-streptavidin conjugated iNKT-TCR tetramers (4C12 and 4C1369) were carried out in the same way as CD1d-tetramer stainings.

### Generation of Soluble Heterodimeric TCRs

Soluble TCR heterodimers were generated as previously described [41]. Briefly, the extracellular region of each TCR chain was individually cloned in the bacterial expression vector pGMT7 and expressed in *Escherichia coli* BL21-DE3 (pLysS). Residues Thr48 and Ser57, respectively, of the α- and β-chain TCR constant region domains were both mutated to cysteine. Expression, refolding, and purification of the resultant disulfide-linked iNKT TCR αβ heterodimers was carried out as previously described [32].

### Surface Plasmon Resonance

Streptavidin (∼5,000 RU) was linked to a Biacore CM-5 chip (BIAcore AB, UK) using the amino-coupling kit according to manufacturer's instructions, and lipid-CD1d complexes or control proteins (βGC-CD1b and HLA-A2*01-NY-Eso-1(157-165) complex) were flowed over individual flow cells at ∼50 µg/ml until the response measured ∼1,000 RU. Serial dilutions of recombinant iNKT TCRs were then flowed over the relevant flow cells at a rate of 5 µl/min (for equilibrium binding measurements) or 50 µl/min (for kinetic measurements). Responses were recorded in real time on a Biacore 3000 machine at 25°C, and data were analyzed using BIAevaluation software (Biacore, Sweden). Equilibrium dissociation constants (KD values) were determined assuming a 1∶1 interaction (A+B ↔ AB) by plotting specific equilibrium binding responses against protein concentrations followed by non-linear least squares fitting of the Langmuir binding equation, *AB = B×AB_max_/(K_D_+B)*, and were confirmed by linear Scatchard plot analysis using Origin 6.0 software (Microcal, USA). Kinetic binding parameters (k_on_ and k_off_) were determined using BIAevaluation software.

### Generation of Stable T2-CD1d Lymphoblast Lines

Stable human CD1d-expressing T2-lymphoblast lines and clones (T2-CD1d) were generated by spin infection of T2 lymphoblasts with lentiviral particles encoding the human CD1d gene. VSV–G pseudotyped lentiviral particles were generated as previously described [42]. The following primers were used to clone full-length human CD1d into the lentiviral vector pHR'SIN18: 5′-AGCGGGATCCGCCGCCACCATGGGGTGCCTGCTGTTTCTGCTG-3′ (forward), and 5′-GCGTCTCGAGTCACAGGACGCCCTGATAGGAAGTTTG-3′ (reverse). In brief, HEK293T cells were co-transfected with 5 µg of pVSV-G [43], 10 µg of the packaging plasmid pCMV δ8.91 [44], and 15 µg of the human CD1d-encoding transfer vector pHR'SIN18-hCD1d by calcium phosphate method. Viral supernatants were harvested 48–60 h post-transfection, filtered, and concentrated by centrifugation at 25,000 rpm, 4°C for 90 min. Viral pellets were resuspended in 1 ml fresh RPMI 1640 for transduction. Transduced cells were maintained in growth medium for 10 d before sorting of human CD1d-expressing T2 single cells and lines on a FACSVantage SE apparatus (Becton Dickinson, USA), using PE-conjugated anti-human-CD1d antibody CD1d42 (Pharmingen, Switzerland).

### Proliferation and Cytokine Secretion Assays

T2 lymphoblast cells (T2-) and CD1d-expressing T2 lymphoblast cells (T2-CD1d) were used as antigen presenting cells (APC). 5×10^4^ iNKT cells were plated in a 96-well round-bottom plate in triplicates with either medium alone, with 2.5×10^4^ T2-CD1d, or with T2 lymphoblasts. Before use, T2-CD1d and T2 lymphoblasts were treated with 0.1 mg/ml mitomycin C for 1 h at 37°C and extensively washed with PBS. Lipid antigens (K7, OCH, and βGC) were added at a final concentration of 100 ng/ml. Lipids were solubilized at 200 µg/ml by sonication in vehicle (0.5% Tween-20), which was also used as a negative control. IL-2 was added to the culture medium at a final concentration of 10 IU/ml. Proliferation was measured during the last 18 h of a 96 h incubation by addition of 1 µCi [^3^H]-methyl-thymidine (1 Ci = 37 GBq, Amersham Pharmacia), followed by harvesting and scintillation counting (Perkin Elmer beta counter).

Levels of IL-4, IL-5, IL-10, IL-13, GM-CSF, IFN-γ, and TNF-α were measured in the cell supernatants, collected after 48 h of incubation, by Bio-Plex suspension array system (Bio-Rad, USA), according to manufacturer's recommendations.

### Cytotoxicity Assays

T2 lymphoblasts and T2-CD1d were cultured for 16 h either in the presence of lipid antigens at 100 ng/ml concentration or an equivalent quantity of vehicle. They were then labeled with 100 µCi of ^51^Cr (GE Healthcare, UK) for 1 h at 37°C and washed 3 times with warm RPMI 1640 supplemented with 1% FBS.

iNKT cells were added in duplicates at different effector-to-target cell ratios and cultured for 4 h. Maximal ^51^Cr release was determined from target cells lysed by hydrochloric acid. The percentage of specific lysis was calculated by the following formula: [(experimental cpm − spontaneous release cpm)/(maximum release cpm − spontaneous release cpm)] ×100%. Percentage of unspecific lysis was always <20%.

### Generation of Fluorescent iNKT TCR Tetramers

Soluble iNKT-TCR heterodimers were biotinylated via an engineered BirA motif on the C-terminus of their TCR β-chain and then conjugated to PE-streptavidin (Molecular Probes, USA). Multimeric complexes were purified by FPLC (Pharmacia, Sweden) on an SD200 column (Pharmacia, Sweden) and concentrated to 1 mg/ml using Vivaspin20 concentrators (Vivascience, UK).

## Abbreviations

βGC: β-glucosylceramide
CDR: complementarity determining region
iNKT: Invariant Natural Killer T-lymphocytes
K7: KRN7000 α-galactosylceramide
LacCer: Lactosylceramide
MFI: mean fluorescent intensity
PBMC: peripheral blood mononuclear cell
